# Optimization of biodiesel synthesis from *Jatropha curcas* oil using kaolin derived zeolite Na–X as a catalyst

**DOI:** 10.1039/d2ra03278c

**Published:** 2022-08-15

**Authors:** Stephen Otieno, Fredrick Kengara, Chrispin Kowenje, Robert Mokaya

**Affiliations:** Department of Chemistry, Maseno University P. O. Box 333-40105 Maseno Kenya; School of Pure and Applied Sciences, Bomet University College P. O. Box 701-20400 Bomet Kenya; School of Chemistry, University of Nottingham, University Park Nottingham NG7 2RD UK r.mokaya@nottingham.ac.uk ckowenje@maseno.ac.ke

## Abstract

Biodiesel is an alternative renewable green fuel obtainable from the reaction of plant or animal oil with a low molecular weight alcohol in the presence of a catalyst. However, the cost of its production remains high due to costly feedstock, the majority of which is competitively also used as food, and the use of homogeneous catalysts, which pose difficulties in product purification and resulting environmental pollution. The aim of this study was to explore the production of biodiesel through transesterification of non-edible and cheap *Jatropha curcas* (JC) oil using a zeolite Na–X catalyst obtained from naturally occurring kaolin clay. The transesterification parameters, namely reaction temperature, reaction time, catalyst loading and methanol to oil molar ratio were optimized using the L_16_(4^4^) Taguchi orthogonal array approach. The catalyst loading was found to be the most influential parameter at 93.79%. The optimum conditions for the conversion of JC oil, with a biodiesel yield of up to 93.94%, were found to be a methanol to oil molar ratio of 10 : 1, catalyst loading of 8%, reaction temperature of 70 °C and reaction time of 5 h. Fuel characterization parameters were within the European Norm (EN) 14214:2019 biodiesel specifications. Our findings offer insights into the ideal parametric conditions for the cost-effective synthesis of biodiesel from JC oil *via* zeolite-catalyzed esterification.

## Abstract 2

Zeolite Na–X derived from inexpensive kaolin clay is an effective catalyst (up to 94% yield) for *Jatropha curcas* oil conversion to biodiesel; Taguchi optimization shows methanol/oil ratio of 10, with 8% catalyst loading at 70 °C for 5 h as optimum.

## Graphical abstract

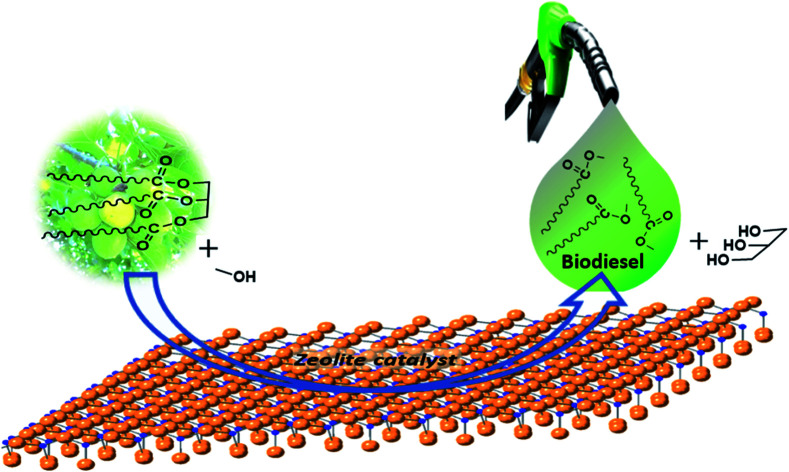

## 1. Introduction

About 80% of current global energy use is sourced from non-renewable fossil fuels, namely natural gas, coal and petroleum oil, which have emissions that have adverse impacts on the environment and health.^1,2^ Biodiesel is amongst the viable alternative biofuels currently in use. Biodiesel is a mixture of fatty acid alkyl esters, derived from a fatty acid chain of a plant or animal oil, and a low molecular weight alcohol, usually methanol and ethanol. Biodiesel was first explored in the late 1970s, but it is only more recently that it has received a great deal of interest.^3^ The current industrial production of biodiesel is expensive because of the use of homogeneous catalysts, which pose potential environmental contamination risks, and the use of edible oils, which are also competitively used as human and animal food. At least 12% of the global edible oil yield is currently used in the production of biodiesel.^4^ About 73% of global biodiesel production is based on vegetable oils (30% soybean oil, 25% palm oil and 18% rapeseed oil) or waste cooking oils (22%).^5^ The use of sustainable and non-edible oils of low direct commercial value to humans, and heterogeneous catalysts, should therefore be explored as alternatives for biodiesel production. From a processing viewpoint, the use of heterogeneous catalysts would drastically reduce the cost of production and, subsequently, the cost of biodiesel.

*Jatropha curcas* is a deciduous plant whose seeds are rich in non-edible oil and which can be sustainably used as a suitable non-food substitute for biodiesel production. The *Jatropha* seeds have an oil content of over 50 wt%,^6^ which can be easily extracted. The presence of toxic phorbol esters renders JC oils non-edible to humans and animals.^7^ JC oils are high in palmitic, oleic and linoleic acids, and more than 75% of the oil is unsaturated fatty acids.^8^ The composition of fatty acids generally depends on the maturity stage of the plant, environmental conditions, and genetics.^9^ Various solid catalysts have been explored as alternatives to current homogenous catalysts,^10–12^ but findings so far indicate a myriad of challenges including catalyst cost, limitations of access to active sites, mass transfer issues, catalyst stability and regeneration. Given these challenges that face utilization of heterogeneous catalysts in the transesterification and esterification reactions, zeolites have come to be viewed as potential panaceas to these limitations. However, the potential of zeolites to overcome the limitations faced by conventional heterogenous catalysts is yet to be established.

Zeolites are crystalline porous aluminosilicate materials built from an infinitely extending network of silicate [SiO_4_]^4−^ and aluminate [AlO_4_]^5−^ tetrahedra, linked to each other by corner sharing of oxygen atoms. The zeolite skeleton supplies active catalytic sites,^13^ thus providing reactive centers for chemical reactions. In addition, the zeolite pore structure optimizes the available surface area. The pores also make it possible for the occlusion of guest species in zeolites. Although zeolite catalysts perform better than metal catalysts,^14^ zeolites in their pure form show very little or no activity towards oil conversion to biodiesel,^15^ and require modification with cations to engender catalytic activity. In modifying zeolites, alkali hydroxides have been used to load metal ions into the zeolite pores, but this can led to structural collapse and subsequent loss of active sites.^16^ Milder modification conditions using alkali salts are therefore more suitable, and, therefore, alkali acetates, chlorides and nitrates are mostly used and can additionally result in high ester yields.^17–19^

The pore size and pore size distribution of zeolites are important considerations as they play a significant role in the mass transfer process during catalytic processes. Đặng and co-workers obtained a high yield of biodiesel using zeolite A synthesized from kaolin but, however, the catalyst load of 72 wt% of triolein was very high and not sustainable.^20^ Furthermore, zeolite A has small pore channels and, therefore, the mass transfer of lipids is likely a limiting factor. Small pore channels are a limiting factor in the zeolite catalysis of oils due to limitations in mass transfer of triglycerides.^21,22^ Zeolite catalysts, by their nature, are dependent on porosity and the hydrophobic/hydrophilic balance in determining interactions with catalytic reactants and products.^23^ It is also known that the yield of fatty acid methyl esters (FAMEs) rises with increase in the number of cations occluded in the zeolite super cages.^14,17,24^

In the transesterification of oils, the reaction parameters – temperature, duration, catalyst concentration, stoichiometry of reactants – affect the catalytic process.^19,25–28^ Optimization of these parameters is necessary in any efforts to maximize the production of desired oils. Given that the optimum transesterification conditions reported in literature vary from study to study,^14,15,18,19,25,26,29^ this study aimed to clarify on the optimum conditions for biodiesel production but also utilized a sustainably obtained zeolite as catalyst. Large pore zeolite Na–X, synthesized from natural kaolin material, was modified through incipient wetness impregnation with CH_3_COONa. The resulting zeolite catalyst was tested in a batch reaction for conversion of JC oil to biodiesel under various transesterification conditions. The transesterification reaction was optimized using the Taguchi orthogonal array method. The findings of this study will inform on the choice of optimum transesterification processes conditions that may be applied for zeolite catalysis of the conversion of JC oil to biodiesel.

## 2. Materials and methods

### 2.1. Materials

The zeolite NA–X used in this work was synthesized using raw kaolin powder, supplied by the Department of Inorganic Chemistry – University of Yaoundé 1, and following previously reported procedures.^30^*Jatropha curcas* seeds were obtained from Dala Rieko in Asembo Bay, Kisumu County – Kenya (0°11′14.2′′S 34°23′12.1′′E). All other chemicals and reagents used were of analytical grade, unless otherwise specified. Sodium hydroxide (NaOH) pellets 99%, *n*-hexane ≥ 95%, *n*-heptane ≥ 99.9%, methanol ≥ 99.9%, chloroform ≥ 99%, magnesium sulfate (drying agent) and anhydrous sodium acetate (CH_3_COONa) ≥ 99.9% were obtained from Fisher Scientific. Supelco fatty acid methyl ester (FAME) mix GLC-10 reference standard and methyl nonadecanoate, ≥98%, internal standard were obtained from Sigma Aldrich. 1.3 mm Whatman 0.45 μm PTEF membrane filters were obtained from GE Healthcare. Deionized water was prepared using an Elga PURELAB Option 4463 water deionizer.

### 2.2. Preparation of zeolite catalyst

Modification of the zeolite Na–X was achieved *via* incipient wetness impregnation with sodium acetate. 1 g of zeolite was mixed with 2 mL of 1.46 M CH_3_COONa solution and the mixture stirred at 600 rpm at 65 °C for 4 h. The calculation of the cation concentration was based on the fact that 1 g of anhydrous zeolite Na–X, (Na_86_(AlO_2_)_86_(SiO_2_)_106_), contains 3.38 × 10^19^ unit cells, with 8 supercages per unit cell, and therefore a total of 2.7 × 10^20^ supercages.^31,32^ This allowed for the calculation of theoretical cation exchange for each unit cell and therefore the theoretical quantity of Na^+^ ions required to compensate the negative charge associated with AlO_2_ units.

The resulting Na-containing zeolite was then vacuum dried at room temperature for 24 h before further drying in the oven at 100 °C for 24 h. The sodium salt in the modified zeolite was decomposed by heating the dry modified zeolites in air at 5 °C min^−1^ to 500 °C, and then holding at this temperature for 4 h to generate Na_2_O occluded zeolite (Na/Na–X zeolite). Prior to use as catalyst, the calcined Na/Na–X zeolite was outgassed by heating at 250 °C for 20 min. In a box furnace, followed by cooling in a desiccator under vacuum.

#### 2.2.1. Characterization of zeolite catalyst

Powder XRD patterns were recorded in a continuous mode over the 2*θ* scan range from 5° to 50° in steps of 0.026° at room temperature using a PANalytical X-Pert Pro X-ray powder diffractometer employing Cu-Kα radiation. X-rays were generated from the Cu anode at 40 kV and a current of 40 mA. The presence of functional groups in the zeolites was monitored by infrared spectroscopy between 400–4000 cm^−1^ using a Bruker Alpha Attenuated Total Reflectance – Fourier Transform Infrared (ATR-FTIR) spectrometer. Thermal properties of the catalysts were investigated through thermogravimetric analysis (TGA) using a TA Instruments SDT Q600 thermal analyzer by heating samples at 10 °C min^−1^ under flowing air (100 mL min^−1^) up to 1000 °C. The morphology of the samples was explored *via* scanning electron microscopy (SEM) using a FEI Quanta 200 3D Dual Beam FIB microscope. Elemental composition was obtained using an energy dispersive spectrometer (EDS) during SEM analysis. The textural properties were obtained using a Micromeritics 3FLEX sorptometer employing N_2_ gas as a sorbate at liquid nitrogen temperature (−196 °C). Samples were outgassed under vacuum at 300 °C for 16 h prior to analysis. The surface area was calculated using the Brunauer–Emmett–Teller (BET) method, and the total pore volume was deduced from the nitrogen uptake at close to saturation pressure (*P*/*P*_0_ ∼ 0.99). Micropore surface area, external surface area and micropore volume were determined from *t*-plot analysis, while pore distribution and pore size were determined by the Horvath–Kawazoe method.^33^

### 2.3. Preparation of *Jatropha curcas* oil

Jatropha seed kennels were obtained by manually removing the seed husks. The seed kennels were then washed with deionized water, dried in air for 24 h and then crushed in a blender to powder, before further drying in the oven at 100 °C for 4 h. The dry seed powder was soaked in *n*-hexane (at a ratio of 1 g of seed powder/2 mL of hexane) in a sealed 2.5 L Winchester glass bottle for 24 h. The seed fiber was filtered off and rinsed with extra *n*-hexane. The *n*-hexane solvent was separated *via* rotary vaporization in vacuum at 32 °C to recover the extracted oil. The oil was placed in a tightly sealed container before storing below 4 °C to avoid rancidity.^34^

### 2.4. Transesterification of *Jatropha curcas* oil

Transesterification, *i.e.*, biodiesel production, with *Jatropha curcas* oil and methanol was performed in a batch process in the presence of zeolite catalysts. The biodiesel synthesis and preparation procedure reported by the Laboratory Analytical Procedure, developed by the National Renewable Energy Laboratory (NREL),^35^ was used with some modifications. In brief, the required amount of catalyst was placed in a 10 mL round bottomed flask and evacuated by heating in a box furnace at 250 °C for 20 min. Before cooling under vacuum as described above. The required amount of JC oil and methanol was then added into the flask, followed by 5 mL of 10 mg mL^−1^ methyl nonadecanoate (C19:0Me) internal standard in heptane.

The transesterification was performed by refluxing the reaction flask content in an oil bath with magnetic stirring at 600 rpm. At the end of the set reaction time, the products were cooled to room temperature. 1 mL chloroform, 3 mL heptane and 3 mL deionized water were then added to the reaction flask, which was then stoppered, vortexed for 15 s, and allowed to settle and separate for 1 h. To the organic layer, 2 g of freshly dried MgSO_4_ was added and the content vortexed before allowing it to stand in a sealed vial for 3 h. The dry organic layer was then withdrawn using a 2 mL plastic syringe and filtered through a 0.45 μm PTFE membrane filter into a 2 mL GC vial in readiness for analysis. The vials were stored at −20 °C.

#### 2.4.1. Optimization of the transesterification reaction by Taguchi method

##### 2.4.1.1. Experimental design array

The optimal transesterification conditions were achieved using an experimental design where a scheme of experiments under varying conditions was performed using Taguchi experimental design. The Taguchi method is a statistical experimental design approach for multifactor process optimization.^36^ It is a fractional factorial design of experiments based on orthogonal arrays, which allows one to evaluate the maximum number of effects from a minimum number of runs in an experiment while allowing for differences in the number of factor levels.^37^ A four level (L_16_(4^4^)) Taguchi orthogonal array design of experiments, with a total of 16 experimental runs, was developed in Minitab 18.1.0.0 software. Table 1 shows the levels of chosen independent factors. Detailed L_16_(4^4^) experimental design matrix used in the optimization study are described in more detail below (Results and discussion Section 3.2.2).

**Table 1:** Levels of chosen independent factors in the L_16_ (4^4^) Taguchi design of experiments

Factors	Level			
	Low		High	
Temperature (°C)	50	60	70	80
Oil/methanol ratio (mol/mol)	1 : 5	1 : 10	1 : 15	1 : 20
Time (h)	0.5	2	5	8
Catalyst load (wt% on wt of oil)	0.5	2	5	8

##### 2.4.1.2. Analysis of variance (ANOVA)

Taguchi method leads to the determination of the factors affecting product quality. The signal to noise (S/N) ratio is used as a loss function to measure the quality characteristics deviating from the desired value. Based on the S/N ratio, it is possible to get the optimum level of the individual process parameters providing the highest yield of biodiesel. In this study, ‘‘larger is better’’ S/N ratio, formula shown in eqn (1), was selected to attain maximum yield of biodiesel.1
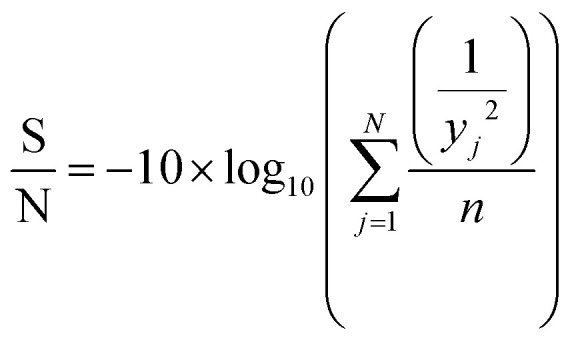
where *y*_*j*_ is the mean value of response (FAME yield), *j* is the trial number and *n* is the number of repetitions of each experiment. The 
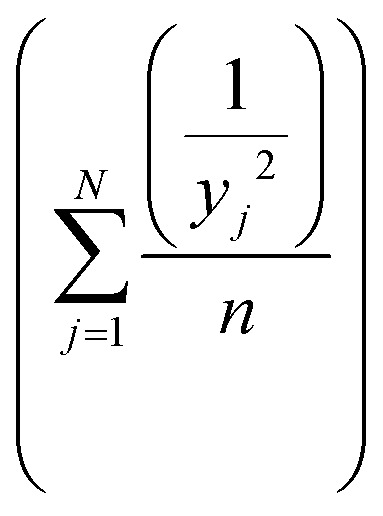
 term is the mean square deviation.

The analysis of variance provides statistical parameters for analysis of the significance of every transesterification parameter and their impacts on the FAME yield. To identify the factor with the most significant effect on the FAME yield and the response magnitude, statistical analysis of variance (ANOVA) of the response data was used. The basic property in ANOVA is that the total variation is equal to the sum of the squares of the deviations (ss) of all the condition parameters and the error components.^38^ The percentage of contribution of the factors was evaluated according to eqn (2).2
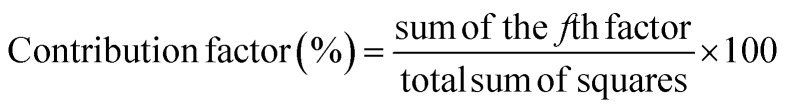


Besides, the Fischer's test (*F*-test) value denotes the significance of a particular experimental factor, a high *F*-test value is obtained when the experimental data fits well with the model chosen or when the noise is high.^39^ The high *F*-test value is verified by the probability value (*P*-value). A *P*-value below 0.05 shows that *F*-value is due to good fit of the model rather than to noise.

### 2.5. Characterization of *Jatropha curcas* oil and biodiesel product

The organic purity analysis (*i.e.* absence of inorganic matter) of oil was performed using thermogravimetric analysis (TGA) as described above.

#### 2.5.1. Determination of response factor (RF)

Prior to analysis of JC oil and biodiesel samples, the response factors (RFs) of individual FAMEs in the supelco multicomponent (GLC-10) reference standard were determined. GLC-10 consists of 20 wt% each of methyl palmitate (C16:0Me), methyl stearate (C18:0Me), methyl oleate (C18:1Me), methyl linoleate (C18:2Me) and methyl linolenate (C18:3Me). The determination of RF was achieved by generating the calibration curves for each FAME component of the GLC-10 reference standard and following the method developed by Wychen *et al.* 2016.^35^ Individual FAME peaks of the GLC-10 reference standard were identified by their retention time. Table 2 shows the five levels of reference standards for generating calibration curves for use in the calculation of RF.

**Table 2:** Five levels of reference standards for the determination of RF^a^

Ref. std (r.s) level	[r.s] (mg mL^−1^)	[i.s] (mg mL^−1^)
1	0.0099	0.025
2	0.0298	0.025
3	0.0994	0.025
4	0.2485	0.025
5	0.497	0.025

a [r.s] is the concentration of the reference standard, [i.s] is the concentration of the internal standard.

The RFs were calculated using the formula given in eqn (3);3
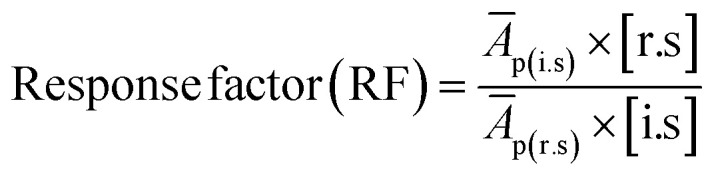
where (*Ā*_p(r.s)_ is the average peak area of individual FAME in the reference standard, *Ā*_p(i.s)_ is the average peak area of the internal standard, [i.s] is the concentration of internal standard, while [r.s] is the concentration of reference standard (components of GLC-10).

On linearization of eqn (3), the response factor is calculated using the formula;4
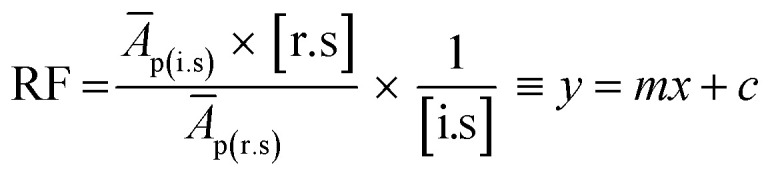

5 RF = slope^−1^ × [i.s]^−1^

where 
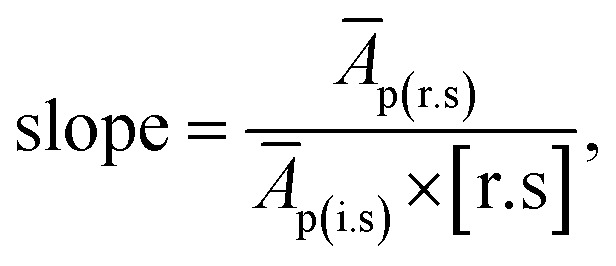
*i.e.*, slope of 
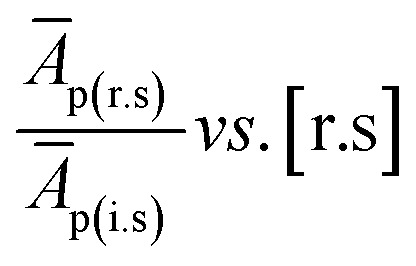


#### 2.5.2. Determination of *Jatropha curcas* oil and biodiesel properties

##### 2.5.2.1. Determination of fatty acid methyl esters composition

The FAME content of transesterification products was determined by gas chromatography (Trace 1310, Thermo Scientific) fitted with a flame ionization detector and SGE BP1 column (30 m × 0.25 mm × 0.25 μm). Helium was used as a carrier gas at 1 mL min^−1^. The optimal conditions for the GC-FID were obtained as; inlet temperature of 250 °C with a 10 : 1 split auto injection of 1 μL, and oven temperature program with initial temperature at 120 °C for 1 min and then ramped at 6 °C min^−1^ to 250 °C followed by holding at this temperature for 5 min. The detector temperature was 280 °C with a hydrogen gas flow rate at 40 mL min^−1^. The GC-FID optimal conditions were based on the best separation achieved for C18:0Me, C18:1Me and C18:2Me peaks of the GLC-10 reference standard.

The content of individual FAME in the biodiesel sample was calculated by the formula;

6 FAME = [i.s] × *A*_p(ME)_ × RF_ME_/*A*_p(i.s)_

where FAME = fatty acid methyl ester content of individual fatty acid, *A*_p(ME)_ is the peak area of the individual methyl ester in the sample, and RF_ME_ is the response factor of the respective reference standard calculated using eqn (5).

The total FAME yield (%) of a biodiesel sample from individual reactions was then calculated using eqn (7).7
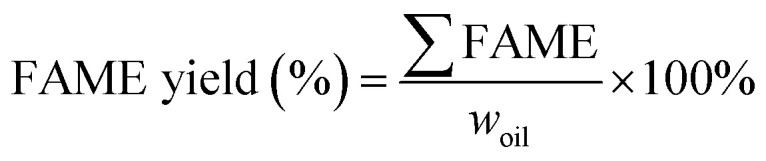
where ∑FAME is the weight summation of fatty acid methyl ester content of individual fatty acids in the biodiesel sample, while *w*_oil_ is the dry weight of the oil sample used in the transesterification reaction. All the experiments were carried out in triplicate and mean values of response were used for further analysis.

##### 2.5.2.2. Determination of other properties of *Jatropha curcas* oil and biodiesel

The vibrational characteristics and determination of the functional groups of *Jatropha curcas* oils and the derived biodiesel and glycerol products was done *via* FTIR spectroscopy. Cetane number (CN) is one of the important properties in evaluating the ignition properties of biofuel because it represents the time delay between the start of injection and the point where the fuel ignites. Cetane number of the derived biodiesel was determined using eqn (8) developed by Mishra and co-workers.^40^ The Cetane number correlation equation includes the interaction effects of straight-chain saturated factor (SCSF) and modified degree of unsaturation (DU_m_).

8 CN = *K*_1_ + (*K*_2_ × DU_m_) + (*K*_3_ × SCSF) + (*K*_4_ × DU_m_ × SCSF)

where; CN = Cetane number, SCSF = straight-chain saturated factor, DU_m_ = modified degree of unsaturation while *K*_1_, *K*_2_, *K*_3_, and *K*_4_ are the correlation constants with values equal to 63.41, −0.073, 0.035 and −3.26 × 10^−4^, respectively.

SCSF is expressed in terms of the molecular weight of saturated methyl esters as;^40^9
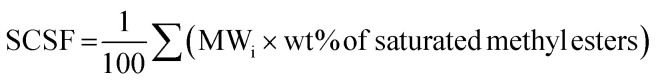
where MW_i_ represents the molecular weight of individual saturated methyl esters.

DU_m_ is expressed in terms of the molecular weight of different unsaturated methyl esters as;^40^10
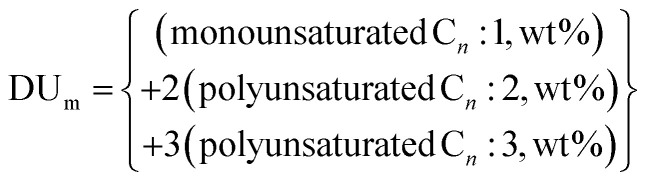


Other properties of JC oil and its transesterification products including density, acid value, viscosity and saponification value were analyzed according to the EN 1421:2012 procedure.^41,42^

## 3. Results and discussion

### 3.1. Characterization of modified zeolite catalysts

**Fig. 1:**
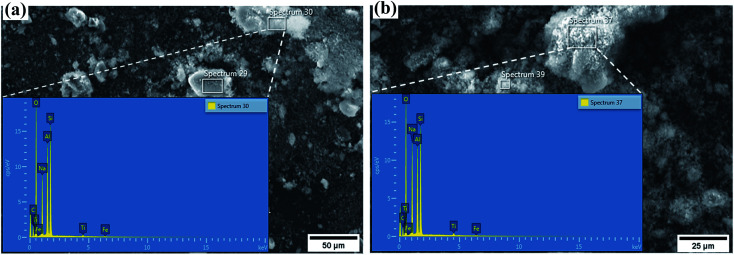
Elemental composition analysis by scanning electron microscopy – Energy dispersive X-ray (SEM-EDX) for, (a) synthesized Na–X (b) Na/Na–X zeolite.

The SEM images and the energy dispersive X-ray spectra obtained during elemental composition analysis of synthesized zeolite Na–X and modified zeolite Na/Na–X are shown in Fig. 1. The chemical compositions are summarized in Table 3. With the exception of Na_2_O, the other oxide's composition is the same for both unmodified and modified zeolite Na–X. The Na_2_O content of the modified zeolite (25.7%) is higher than that of the unmodified zeolite (18.7%) due to successful incorporation of Na^+^ ions into the zeolite structure.

**Table 3:** Chemical composition of unmodified and modified zeolite Na–X

Sample	Percent oxide (wt%)						Calculated chemical formula
	Al_3_O_2_	SiO_2_	Na_2_O	TiO_2_	Fe_2_O_3_	SiO_2_/Al_2_O_3_	
Synthesized Na–X	31.6	48.5	18.7	0.9	0.2	2.60	Fe_0.4_Ti_1.8_Na_97.6_Al_100_Si_130.2_O_465.4_
Na/Na–X	29.1	44.1	25.7	0.8	0.3	2.58	Fe_0.6_Ti_1.8_Na_145.2_Al_100_Si_128.7_O_486.4_

**Fig. 2:**
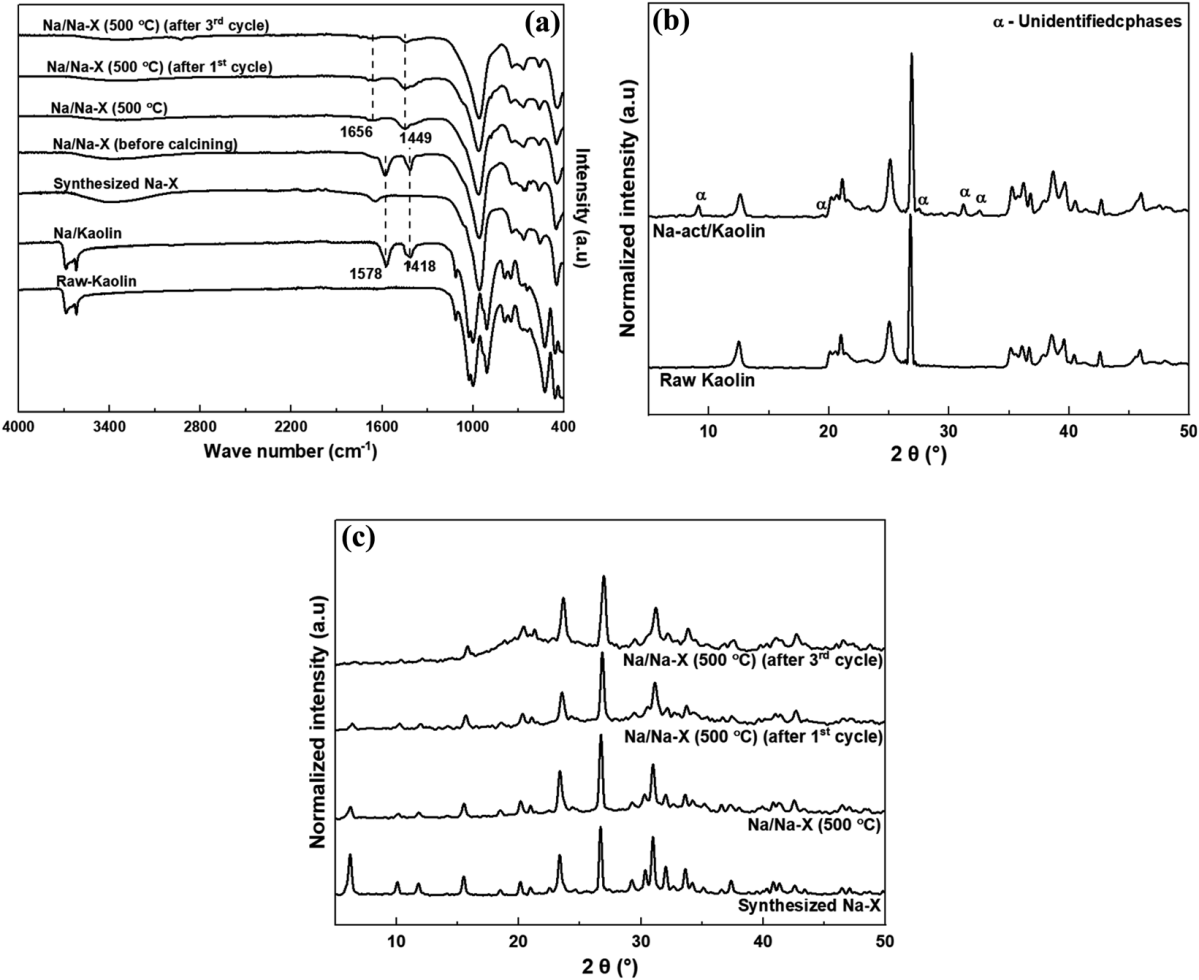
(a) FTIR spectra of kaolin and zeolite Na–X before and after various modifications, (b) XRD patterns of kaolin and kaolin modified with Na-acetate, and (c) XRD patterns of zeolite Na–X before and after various modifications.

The FTIR spectra and XRD patterns of the study materials are shown in Fig. 2. The FTIR spectra (Fig. 2a) indicates the emergence of new hydroxyl bands at 1418 and 1578 cm^−1^ after modification of both Kaolin and zeolite Na–X.^43^ The two bands shifted to higher wave numbers at 1449 and 1656 cm^−1^, but with decreased intensities, after calcining the modified zeolite at 500 °C. The shifts might be due to migration of the hydroxyl groups within the zeolite structure due to heat activation of zeolite. Apart from the diminished spectral bands at 1449 and 1656 cm^−1^ for the catalyst in the subsequent transesterification cycles, other zeolite functional groups were not affected by modification and usage as catalysts, and recycling of the catalyst.

The introduction of Na-acetate onto kaolin results in new phases in the Na-act/kaolin as indicated by the new XRD peaks at 2*θ* of 9.14°, 19.46°, 27.54°, 31.22° and 32.51° in Fig. 2b. As shown in Fig. 2c, modification of the synthesized zeolite Na–X by impregnation with Na-acetate resulted in a reduction in peak intensity. A similar observation was reported by Zhang and Wang for X-type zeolite following Cu loading at amounts higher than 1.0 g L^−1^.^44^ However, it is worth noting that the zeolite's crystallinity was retained after introduction of Na. However, there was observed reduction in the XRD peaks in the low 2*θ* region after the third transesterification reaction cycle of biodiesel synthesis.

**Fig. 3:**
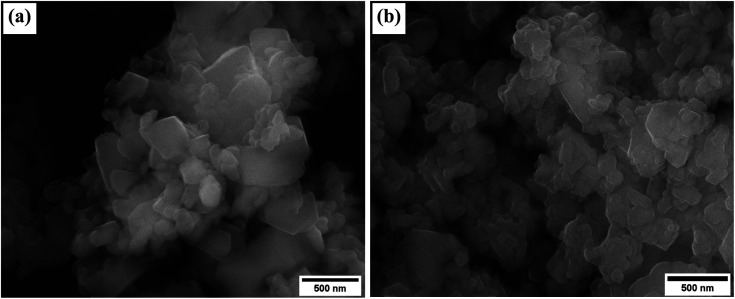
The SEM images of; (a) as synthesized zeolite Na–X, and (b) Na/Na–X zeolite.

The surface morphology of the unmodified and modified zeolite is evident from the SEM images in Fig. 3. Octahedral crystals of varying size that are similar to those previously reported for zeolite Na–X^30^ were observed. The integrity of the crystals was not altered after impregnation with Na-acetate, which is in agreement with observations from the XRD patterns.

**Fig. 4:**
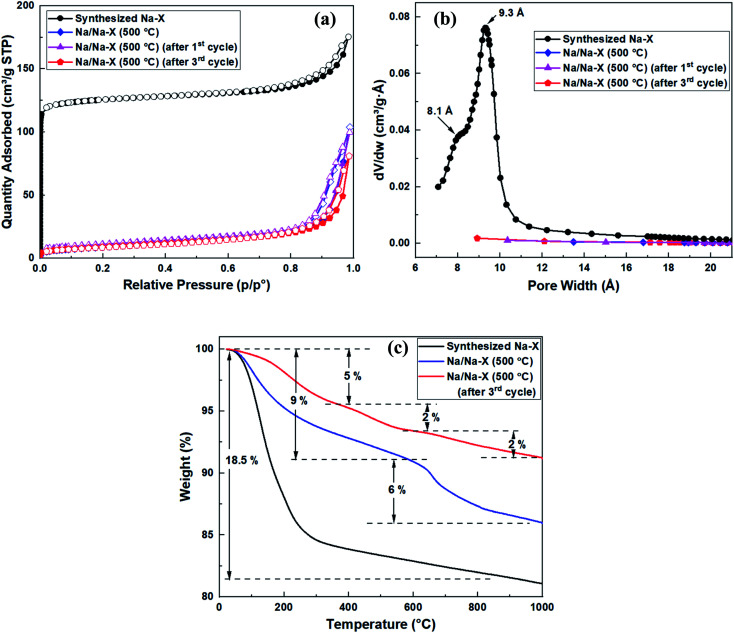
Porosity and thermal analysis of synthesized zeolite Na–X and Na/Na–X zeolite; (a) nitrogen sorption isotherms, (b) Horvath–Kawazoe pore size distribution curves, and (c) TGA curves.

The nitrogen sorption isotherms and textural properties of the modified zeolite Na–X before and after use as catalyst, in comparison to the unmodified zeolite, are shown in Fig. 4 and Table 4. There was a significant reduction in total and micropore surface area from 473 and 429 m^2^ g^−1^ to 39 and 0.6 m^2^ g^−1^, respectively (Fig. 4a). The reduction in surface area is attributed to the presence of Na_2_O clusters and bridges inside the zeolite pores, leading to narrow pores, which hinder N_2_ sorption in the modified zeolite. The narrowing of pores is also evidenced by the pore size distribution curve in Fig. 4b, where no pore maxima is observed for the Na/Na–X zeolites.

**Table 4:** Textural properties of unmodified and modified zeolites

Sample	*S*_(BET)_ (m^2^ g^−1^)	*t*-Plot *S*_(micro)_ (m^2^ g^−1^)	*t*-Plot *S*_(ext)_ (m^2^ g^−1^)	*V*_(total)_ (cm^3^ g^−1^)	*t*-Plot *V*_(micro)_ (cm^3^ g^−1^)	Median pore size (Å)
Synthesized Na–X	473	429	45	0.27	0.18	8.1, 9.3
Na/Na–X (550 °C)	39	0.6	38	0.15	—	—
Na/Na–X (550 °C) (after 1st cycle)	32	—	—	0.16	—	—
Na/Na–X (550 °C) (after 3rd cycle)	32	—	—	0.13	—	—

Thermal gravimetric analysis curves for the modified and unmodified zeolite Na–X are shown in Fig. 4c. An 18.5% weight loss, due to removal of absorbed water, is observed as a single step for the unmodified zeolite Na–X. For the Na/Na–X (500 °C) zeolite, the weight loss occurred in two steps: the first step (at 50–600 °C) involved 9% weight loss due to removal of absorbed water, while the second step (at 600–1000 °C) involved 6% weight loss and may be ascribed to decomposition of the zeolite. On the other hand, three steps of weight loss were observed after the third cycle of catalyst use. The first and second steps (at 50–600 °C) are due to loss of absorbed molecules while the third step (at 600–1000 °C) may be ascribed to catalyst decomposition. The significant reduction in amount of absorbed water for the Na/Na–X zeolites, in comparison to that of zeolite Na–X, is consistent with the trends observed in porosity following Na-acetate impregnation (Fig. 4a and Table 4).

### 3.2. Characterization of *Jatropha curcas* oil and biodiesel

#### 3.2.1. Identification of GC peaks and determination of response factor (RF)

**Fig. 5:**
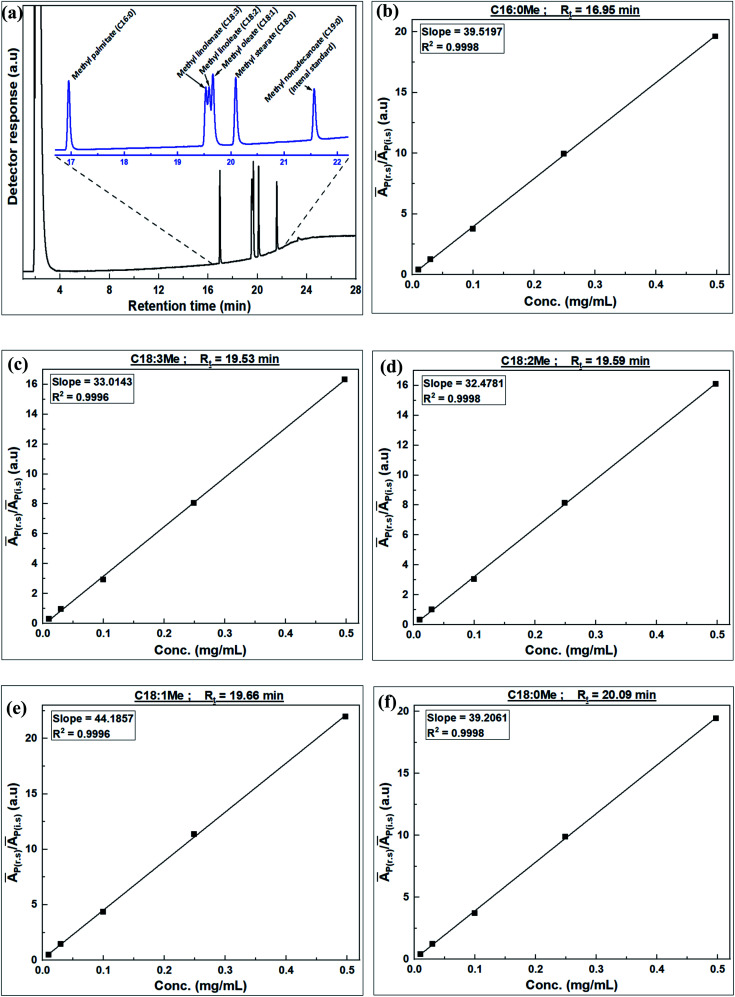
GC trace of Supelco FAME mix GLC-10 reference standard and JC oil FAMEs (a), and plots of the ratio of average peak area of individual FAME to average peak area of internal standard (*Ā*_p(r.s)_/*Ā*_p(i.s)_) *versus* concentration of the FAME in GLC-10 for FAMEs; (b) C16:0Me, (c) C18:0Me, (d) C18:1Me, (e) C18:2Me, and (f) C18:3Me, *R*_t_ = retention time.

GC-FID was used to identify fatty acid components in JC oil (after derivatization to FAMEs) by comparison of retention time with that of the GLC-10 reference standard. Fig. 5 shows the GC trace of the GLC-10 reference standard and JC oil, and the plots used in calculating RF for each FAME component in the GCL-10 reference standard spectrum. The GC-FID response factors for each FAME component, calculated using eqn (5), are summarized in Table 5.

**Table 5:** GC-FID response factors for each FAME component of GLC-10 reference standard

FAME	Retention time, *R*_t_ (min)	Response factor (RF)
Methyl palmitate (C16:0Me)	17.0	1.0122
Methyl linolenate (C18:3Me)	19.5	1.2116
Methyl linoleate (C18:2Me)	19.6	1.2316
Methyl oleate (C18:1Me)	19.7	0.9053
Methyl stearate (C18:0Me)	20.1	1.0202

#### 3.2.2. Characterization of *Jatropha curcas* oil

**Fig. 6:**
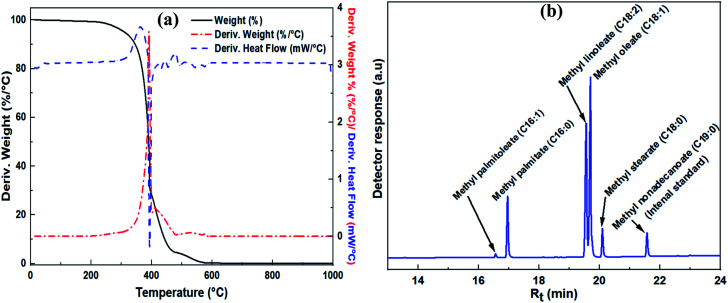
Characterization of JC oil and transesterification products; (a) TGA curve of JC oil, and (b) GC spectra of derived biodiesel.

Thermogravimetric analysis (TGA) curves and GC trace of the JC oil and transesterification products are shown in Fig. 6. Three weight loss steps for the oil, due to carbonization and subsequent burn off of the carbon, were observed as shown in Fig. 6a. The 100% weight loss, for the oil, implies that the JC oil was purely organic with no inorganic content.

The identity of individual FAME components derived from the fatty acids of JC oil are shown in the GC-FID spectrum in Fig. 6b. Individual FAME composition of JC oil, calculated using eqn (6), is given in Table 6. The FAME composition, derived from the fatty acid composition of JC oil, is consistent with that reported in literature.^8,45^ The highest FAME yield from the transesterification of JC oil under optimized conditions was 93.94%.

**Table 6:** Summary of individual FAME components of the JC oil

FAME	Composition (%)
Methyl palmitate (C16:0Me)	13.7
Methyl stearate (C18:0Me)	6.3
Methyl oleate (C18:1Me)	37.2
Methyl linoleate (C18:2Me)	41.2
Others	1.6
Total FAME composition	100

#### 3.2.3. Optimization of transesterification reaction

**Fig. 7:**
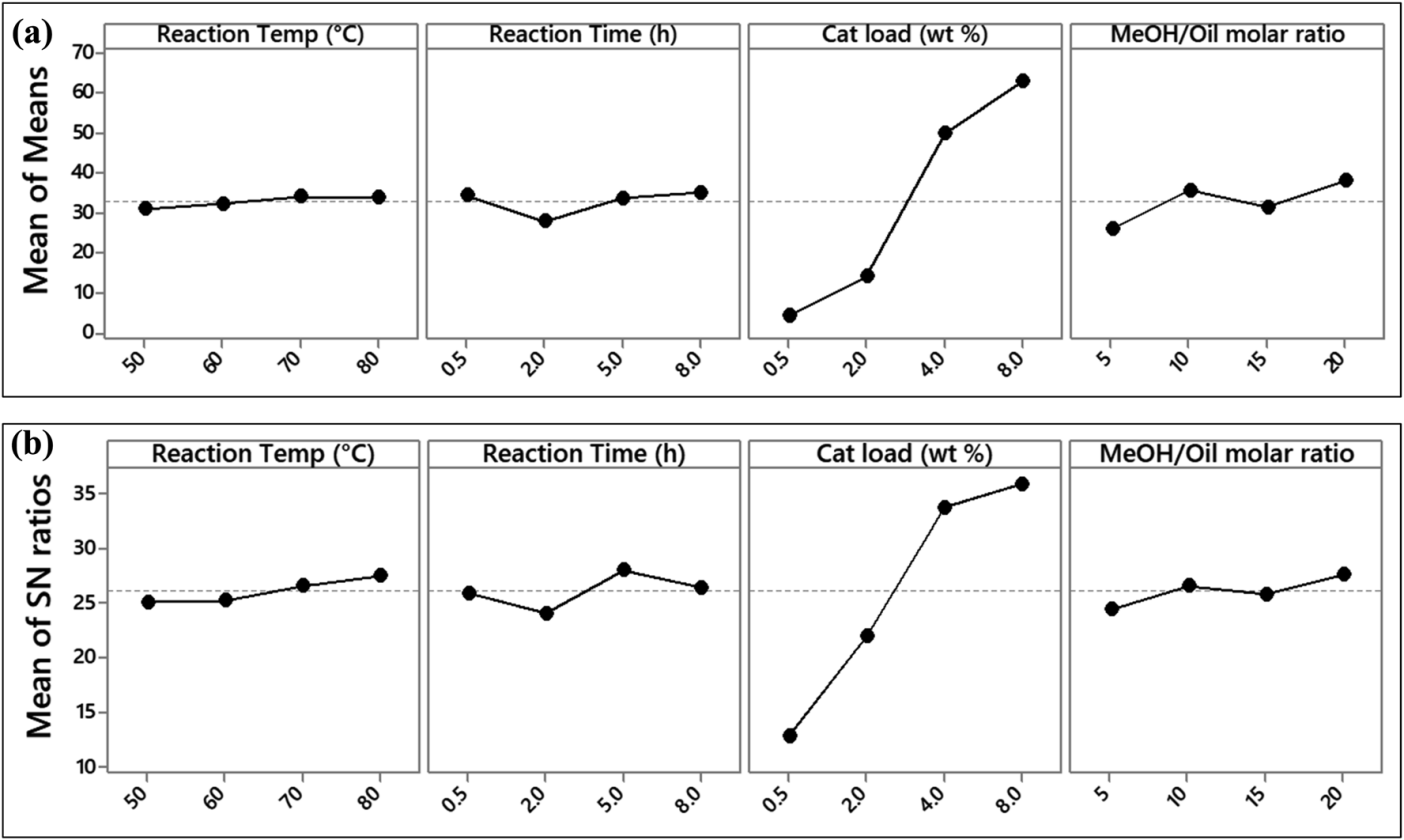
The main effects plot for (a) data mean, (b) S/N ratios for the FAME yield.

Table 7 shows the L_16_(4^4^) experimental design matrix used in this study and the response values (FAME yield (%)), S/N ratio and predicted FAME yield. The main variations with respect to data mean (averages) and S/N ratios, obtained from Table 7, are plotted in Fig. 7.

**Table 7:** The L_16_ (4^4^) experimental design matrix with FAME yield response values and S/N ratio

Experimental run	Reaction temp. (°C)	Reaction time (h)	Catalyst load (wt%)	MeOH/oil molar ratio	Avg FAME content (%)	Std deviation	S/N ratio	Predicted FAME content (%)
1	50	0.5	0.5	5	3.95	0.04	11.92	2.51
2	50	2	2	10	8.80	0.08	18.89	10.45
3	50	5	4	15	46.76	0.17	33.40	47.79
4	50	8	8	20	65.09	0.29	36.27	68.87
5	60	0.5	2	15	10.24	0.26	20.20	14.01
6	60	2	0.5	20	3.31	0.19	10.35	4.33
7	60	5	8	5	55.03	0.57	34.81	56.68
8	60	8	4	10	61.25	0.59	35.74	54.80
9	70	0.5	4	20	56.59	0.32	35.05	58.25
10	70	2	8	15	64.40	0.03	36.20	58.10
11	70	5	0.5	10	5.77	0.18	15.21	9.54
12	70	8	2	5	10.17	0.06	20.14	11.20
13	80	0.5	8	10	67.55	0.71	36.59	68.59
14	80	2	4	5	35.70	0.53	31.05	39.48
15	80	5	2	20	27.97	0.27	28.93	21.51
16	80	8	0.5	15	4.86	0.26	13.71	6.52

From the mean of means and S/N ratios plots in Fig. 7, it is evident that catalyst loading has the strongest influence on the transesterification of JC oil. The highest FAME yield was observed at a catalyst loading of 8%. A higher mean of the control factor is an indication of its stronger effect at that level of FAME yield.^38^ It can also be seen that for catalyst loading, the increase in FAME yield was very steep, in comparison to other parameters where the rise was much lower. The ranking of parameters tested from the S/N ratio response (Table 8) also show that catalyst loading had the highest influence on FAME yield, followed by reaction time, methanol to oil molar ratio, and finally the reaction temperature. The rankings are based on the delta values, *i.e.*, the difference between the highest and the lowest signal to noise ratio level.^46^

**Table 8:** Response table for signal to noise ratios

Level	Reaction temp (°C)	Reaction time (h)	Catalyst loading (wt%)	Methanol/oil molar ratio
1	25.12	25.94	12.80	24.48
2	25.28	24.12	22.04	26.61
3	26.65	28.09	33.81	25.88
4	27.57	26.47	35.97	27.65
Delta	2.45	3.96	23.17	3.17
Rank	4	2	1	3

**Fig. 8:**
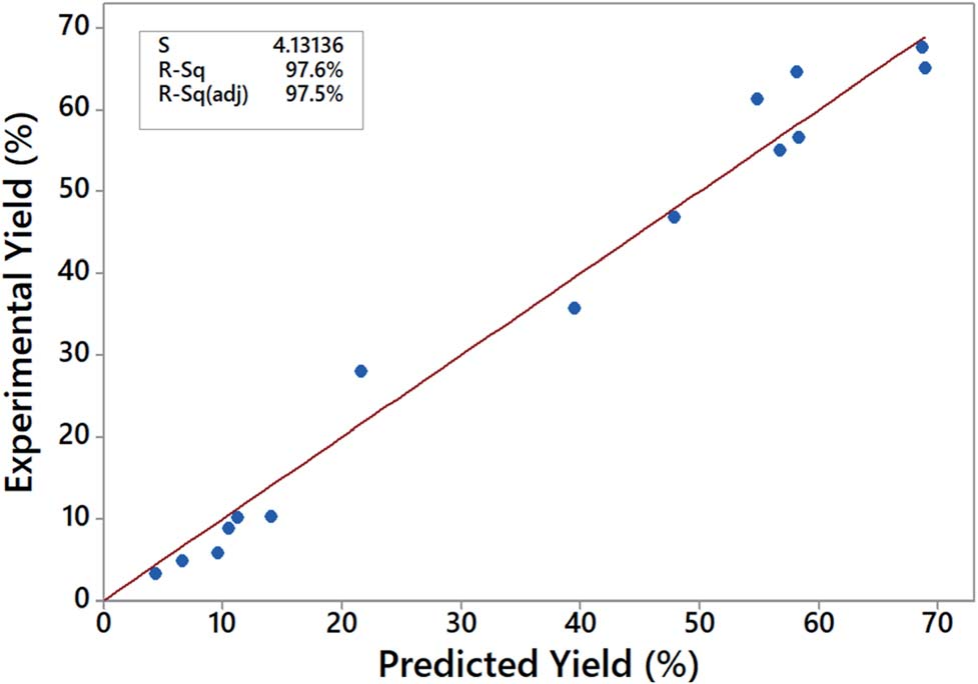
A plot of experimental FAME yield *vs.* predicted FAME yield.

Optimum transesterification conditions depend on the raw materials used and catalyst type, and therefore, the conditions reported for biodiesel synthesis in literature vary from study to study.^17,18,25,26,47,48^ In this work, the optimum conditions for the transesterification reaction of JC oil were found to be 8% by mass of catalyst loading, 5 h reaction time, 10/1 molar ratio of methanol/oil and at 70 °C. To test the accuracy of the chosen model, we plotted the actual yield *versus* predicted yields (data given in Table 7) as shown in Fig. 8. The closeness of points to the 45° regression line and high *R*^2^ values show that the model fitted well with the experimental data. The difference between the *R*^2^ (97.6%) and adjusted *R*^2^ (97.5%) values is also less than the acceptable margin of 0.2,^49^ thereby validating the experimental data.

##### 3.2.3.1. Selection of model optimization parameters

ANOVA results are shown in Table 9. The contribution from the catalyst loading to the transesterification reaction is the most significant at 93.79%. Other factors, namely, reaction time, methanol to oil molar ratio and reaction temperature have much lower significance at 2.14%, 1.43% and 1.10%, respectively. Besides, catalyst loading has the highest *F*-value (60.82) and the least *P*-value (0.003), confirming that it had the highest significance among the four actors considered.^39^ The model fitting is therefore significant, and can be used to optimize the transesterification of JC oil using Na-acetate modified zeolites as catalyst.

**Table 9:** Analysis of variance for SN ratios

Source	DF	Seq SS	Adj SS	Adj MS	*F*	*P*	SS (%)
Reaction temp (°C)	3	16.40	16.40	5.467	0.71	0.607	1.09807
Reaction time (h)	3	32.02	32.02	10.674	1.39	0.397	2.143914
Catalyst load (wt%)	3	1400.78	1400.78	466.927	60.82	0.003	93.78988
MeOH/oil molar ratio	3	21.29	21.29	7.098	0.92	0.525	1.425482
Residual error	3	23.03	23.03	7.678			1.541984
Total	15	1493.53					100

#### 3.2.4. Characterization of biodiesel product

**Fig. 9:**
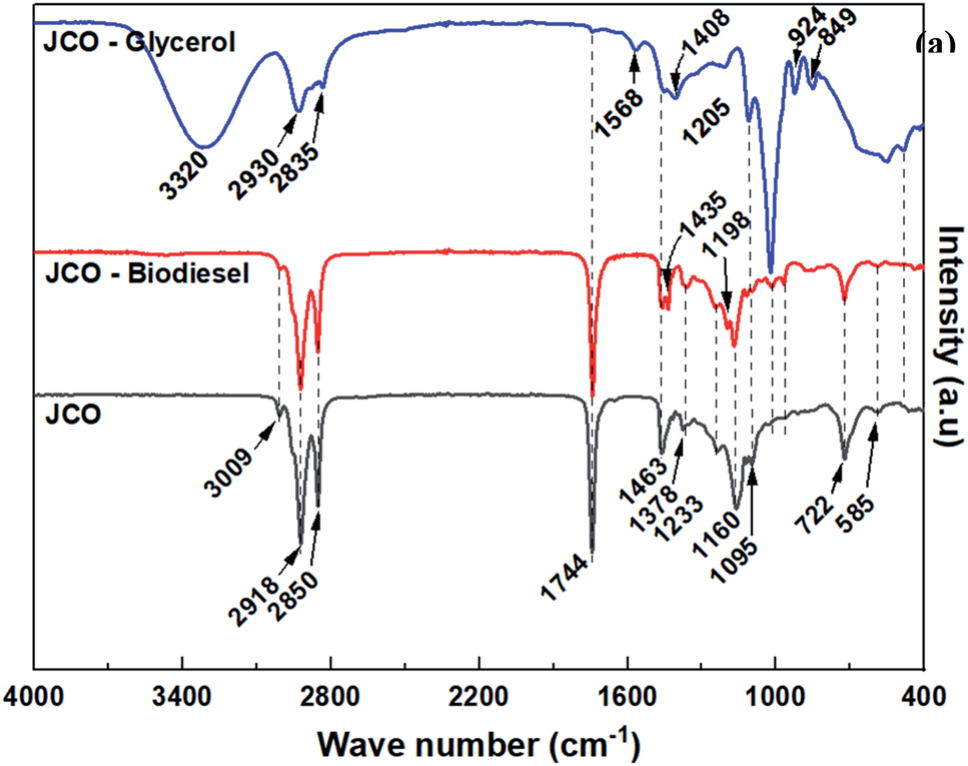
FTIR spectra of *Jatropha curcas* oil and the derived biodiesel and glycerol products.

The FTIR spectra of *Jatropha curcas* oil and the derived biodiesel and glycerol products are shown in Fig. 9. The FTIR spectrum of the oil is expected to be largely similar to that of biodiesel (FAMEs). Small differences are, however, observed in the 400–1500 cm^−1^ region. According to Rafati and co-workers,^50^ the observed bands at 1198 and 1435 cm^−1^ in the spectrum of biodiesel (which are missing in the oil spectrum) are associated with the bending and stretching vibrations of the –OCH_3_ group. In addition, the band at 1160 cm^−1^ in the oil spectrum, which is due to the stretching vibration of the triglyceride C–O group attached to –CH_2_ of the glycerol backbone, slightly shifts to a peak at 1167 cm^−1^ for the FAMEs, corresponding to the stretching vibration of C–O group attached to –CH_3_ of the introduced methanol.

Other observed spectral bands were assigned as follows; the broad band at 3320 and 1568 cm^−1^ (–OH stretching vibration), 3009 cm^−1^ (unsaturated carbon chain =C–H stretching vibration), bands at 2918 cm^−1^ and 2850 cm^−1^ (saturated carbon chain, –CH_2_ symmetrical and asymmetrical stretching vibration), 1744 cm^−1^ (–C=O stretching vibration), 1463 cm^−1^ and 1378 cm^−1^ (bending vibration of –CH_2_ and –CH_3_, respectively), 1223, 1160 and 1095 cm^−1^ (–C–O stretching vibrations) and the band at 722 cm^−1^ (*cis* –CH=CH– out of plane bending vibrations).^51^ The absence of the –OH bands in triglyceride and biodiesel spectra indicates the lack of mono and diglycerides as well as unreacted glycerol and methanol in the oils and biodiesel.^50^

The properties of the JC oil and the derived biodiesel in comparison with the EN 14214:2012,^42^ are shown in Table 10. There is a significant decrease in viscosity and acid value for the transesterification products, compared to biodiesel specifications. The properties of the biodiesel derived from JC oil are, however, consistent with those previously reported for biodiesel.^8,9,52^

**Table 10:** Properties of JC oil and derived biodiesel^a^

Property	*Jatropha curcas* oil	Biodiesel	EU biodiesel specifications, (EN 14214:2012)^42^
Ester content (wt%)	n.a	93.94	96.5
Density (g mL^−1^)	0.92	0.85	0.86–0.90
Kinematic viscosity (mm^2^ s^−1^)	39.63	3.05	3.5–5.0
Iodine value (g_I2_/100 g)	105.01	101.00	≤120
Acid value (mg_KOH_ g^−1^)	4.86	0.50	≤0.50
Saponification value (mg_KOH_ g^−1^)	175.00	n.d	—
Linolenic acid methyl ester (wt%)	n.a	≤1.6	12.0
Cetane number		54.5	≥51.0

a n.d = not done, n.a = not applicable.

Except for the FAME content, all the other tested parameters were within biodiesel specifications. This shows that zeolites derived from less expensive natural clay minerals are promising catalysts for the transesterification of non-edible plant oils with high free fatty acids such as JC oils. It is important to note that the determined CN value is consistent with the reported CN values for similar *Jatropha curcas* biodiesel fuel.^53–55^ The slightly lower ester content of present biodiesel (93.94%) may be an indication of more catalyst loading requirement for higher conversion within the shortest reaction time. Moreover, it may also signify the need for further fine tuning of the zeolite catalysts so as to increase their catalytically active surface area. Table 11 compares the yield of biodiesel from this work with what has previously been reported. The present biodiesel yields compare favourably with previous findings although there are some notable differences. Our biodiesel yield with Na/Na–X as catalyst is slightly lower that K/Na–X is utilised. The other slight differences may arise from variations in zeolite and catalyst preparation methods, oil properties as well as biodiesel preparation conditions and purification. Overall, though, the yield from our Na/Na–X catalyst is in line with biodiesel yields from similar catalysts,^18,56^ but with the added advantage of the catalyst being prepared from low cost naturally occurring kaolin.

**Table 11:** Comparison of current the work to various literature^a^

Catalyst	Catalyst preparation	Transesterification conditions	Oil source	FAME yield (%)	Cycles	Ref.
K/Na–X	Synthesized from rice husk silica	MeOH : oil molar ratio – 16 : 1, catalyst loading of 4 wt%, reaction temperature of 65 °C for 3 h	Jatropha	95.2	—	17
	Wet impregnation with CH_3_COOK					
K/Na–X	Commercial zeolite	MeOH : oil molar ratio of 21 : 1, catalyst loading of 10 wt% and temperature of 60 °C for 6 h	Sunflower	98.2	—	19
	Wet impregnation with KNO_3_					
K/Na–X	Synthesized from coal flyash	MeOH : oil mass ratio of 12 : 1, catalyst loading of 5 wt% and temperature of 65 °C for 7 h	Mustard	84.6	3	26
	Ion-exchanged with CH_3_COOK					
K/Na–X	Commercial zeolite	MeOH : oil mass ratio of 18 : 1, catalyst loading of 12.5 wt% and temperature of 60 °C for 7 h	Safflower	94	3	56
	Impregnation with KNO_3_					
K/Na–X	Synthesized from commercial aluminosilicates	MeOH : oil mass ratio of 15 : 5, catalyst loading of 4 wt% and temperature of 60 °C for 3 h	Palm	97.9	2	57
	Impregnation with CH_3_COOK					
Na/Na–X	Commercial zeolite	MeOH : oil mass ratio of 12 : 1, catalyst loading of 10 wt% and temperature of 60 °C for 5 h	Safflower	98	3	56
	Impregnation with KNO_3_					
Na/Na–X	Commercial zeolite	MeOH : oil molar ratio of 6 : 1, catalyst loading of 10 wt% and temperature of 60 °C for 6 h	Sunflower	93.3	3	18
	Wet impregnation with CH_3_COONa					
Na/Na–X	Synthesized from kaolin	MeOH : oil mass ratio of 10 : 1, catalyst loading of 8 wt% and temperature of 70 °C for 5 h	Jatropha	93.9	3	This work
	Wet impregnation with CH_3_COONa					

a MeOH = methanol.

Regeneration of the catalyst was performed by washing the used zeolite with methanol several times. The washed catalyst was then dried at 100 °C for 12 h before calcining at 500 °C prior to re-use. Biodiesel yields of 91.37% and 88.05% were obtained in the second and third re-use of the zeolite catalysts, respectively. The zeolite catalyst obtained in this study is therefore suitable for use in multiple catalytic transesterifications of the JC oil.

## 4. Conclusions

Sodium containing zeolite catalysts, synthesized from natural kaolin as a source of aluminosilicates, were explored as potential catalysts in the transesterification reaction of non-edible *Jatropha curcas* (JC) oil. Apart from the reduction in XRD intensities for the Na-acetate modified zeolite, the FTIR, XRD and SEM analysis showed that there was no change in crystallinity and morphology. TGA and porosity analysis, however, showed significant reductions in mass loss due to retained water, and to the total and micropore surface area for the Na-acetate modified zeolite. The transesterification parameters were optimized using the Taguchi method while the level of significance of the selected reaction parameters for the transesterification of JC oil was determined using ANOVA. Based on the ANOVA, the most significant parameter in the transesterification of JC oil using the modified zeolite NA-X was found to be the catalyst loading. The optimum conditions for the conversion of JC oil to biodiesel were found to be methanol to oil molar ratio of 10/1, catalyst loading (based on wt of oil) of 8%, reaction temperature of 70 °C and reaction time of 5 h. Apart from a slightly lower the FAME yield, other tested parameters were within accepted biodiesel specifications. Our findings show that zeolites derived from less expensive natural clay minerals are a viable alternative source of aluminosilicates for the preparation of promising catalysts for the transesterification of non-edible plant oils with high free fatty acids such as JC oils to biodiesel.

## Conflicts of interest

There are no conflicts to declare.

## Supplementary Material
